# Supplementary material for: A Case Series and Literature Review of Telogen Effluvium and Alopecia Universalis after the Administration of a Heterologous COVID-19 Vaccine Scheme
Source: Vaccines (Basel). 2023 Feb 15;11(2):444. doi: 10.3390/vaccines11020444 (PMC9968192; doi:10.3390/vaccines11020444)
Supplement: Supplementary file 1 [file vaccines-11-00444-s001.zip › vaccines-2168771-supplementary.pdf]

Supplementary File S1. Literature review of patients who developed Alopecia Areata after COVID-19 vaccination.

Title	Author / Date	n patients	Age / Gender	Ethnicity	Family history	Personal history	Vaccine received	Symptoms	Post-vaccination onset time	Therapy	Evolution
Alopecia areata after ChAdOx1 nCoV-19 vaccine (Oxford/AstraZeneca): a potential triggering factor?	Essam R. et al. / August 2021	1	32 / F	ND	No	AA	AstraZeneca (1st dose)	Head AA	A few days later	ND	ND
Recurrence of alopecia areata after covid-19 vaccination: A report of three cases in Italy	Rossi et / November 2021	3	1) 76 / F	ND	ND	1) AA	1) Pfizer/BioNTech (1st dose)	1) Head AA	1) 2 weeks	ND	ND
			2) 59 / F	ND	ND	2) AA	2) AstraZeneca (1st dose)	2) Head AA	2) 3 weeks	ND	ND
			3) 29 / F	ND	ND	3) AA	3) AstraZeneca (1st dose)	3) Head AA	3) 2 weeks	ND	ND
COVID-19 vaccination and alopecia areata: a case report and literature review	Ganjei Z. et al. / February 2022	2	1) 23 / F	ND	1) No	1) No	1) AstraZeneca (1st dose)	1) Head and eyebrow AA	1) 1 week	1) Betamethasone + Pimecrolimus - Prednisolone	ND
			2) 26 / F	ND	2) No	2) AA	2) AstraZeneca (2nd dose)	2) Head AA	2) 2 weeks	2) Prednisolone	ND
Alopecia areata after SARS-CoV-2 vaccination	Scollan M. et al. / February 2022	9	1) 33 / F	ND	1) AA brother	1) Hepatitis B	1) Modern (2nd dose)	1) Head AA	1) 2 months	1) Tofacitinib	ND
			2) 57 / F	ND	2) No	2) AA	2) Pfizer/BioNTech (2nd dose)	2) Head AA	2) 4 months	2) Tofacitinib	ND
			3) 62 / F	ND	3) No	3) AA	3) Modern (2nd dose)	3) AU	3) 2 months	3) Tofacitinib	ND
			4) 28 / F	ND	4) No	4) AA + Hashimoto's thyroiditis	4) Pfizer/BioNTech (2nd dose)	4) AU	4) 1 week	4) Tofacitinib	ND
			5) 29 / F	ND	5) No	5) Anti Tg and Anti TPO antibodies	5) Pfizer/BioNTech (2nd dose)	5) Head AA	5) 1 week	5) Triamcinolone intra-lesion	ND

			6) 22 / M	ND	6) No	6) Anti Tg and Anti TPO antibodies	6) Moderna (2nd dose)	6) Head and beard AA	6) 1 month	6) Tofacitinib	ND
			7) 15 / M	ND	7) Hashimoto's thyroiditis grandmother	7) No	7) Pfizer/BioNTech (2nd dose)	7) AA head	7) 1 week	7) Triamcinolone intra-lesion	ND
			8) 61 / M	ND	8) No	8) No	8) Pfizer/BioNTech (1st dose)	8) AT	8) 2 weeks	8) None	ND
			9) 16 / M	ND	9) No	9) No	9) Pfizer/BioNTech (1st dose)	9) AA head, eyebrows, and eyelashes	9) 1 to 2 weeks	9) Tofacitinib	ND
Intractable alopecia areata following the second dose of COVID-19 vaccination: Report of two cases	Chen C. et al. / June 2022	2	1) 29 / M	ND	1) No	1) AA	1) AstraZeneca (2nd dose)	1) AA head	1) 1 week	1) Corticosteroid therapy in pulses	No improvement
			2) 26 / F	ND	2) No	2) AA	2) Pfizer/BioNTech (2nd dose)	2) AU	2) 2 weeks	2) Corticosteroid therapy in pulses	No improvement
Alopecia areata after COVID-19 vaccination	Gallo G. et al. / November 2022	1	31 / H	White	No	No	Pfizer/BioNTech (2nd dose)	Head and beard AA	The day after the injection	ND	ND
Induction of alopecia areata after COVID-19 vaccination: a case report in the United States	Lin D. A. et al. / November 2022	1	57 / M	White	ND	Psoriasis + T2DM	Pfizer/BioNTech (1st dose)	Head AA	The day after the injection	Triamcinolone + Mini Dexamethasone Pulses - Clobetasol - Minoxidil	95% regrowth after 8 months

F: female, M: male, AA: alopecia areata, AU: alopecia universalis, AT: alopecia totalis, ND: no data available
